# Adverse effects of hyperbaric oxygen therapy: a systematic review and meta-analysis

**DOI:** 10.3389/fmed.2023.1160774

**Published:** 2023-05-18

**Authors:** Yuyao Zhang, Yijun Zhou, Yuanyuan Jia, Tiantian Wang, Dianhuai Meng

**Affiliations:** ^1^School of Rehabilitation Medicine, Nanjing Medical University, Nanjing, Jiangsu, China; ^2^First Affiliated Hospital, Nanjing Medical University, Nanjing, Jiangsu, China

**Keywords:** randomized controlled trial, systematic review and meta-analysis, hyperbaric oxygen therapy, adverse effects, safety

## Abstract

**Introduction:**

Hyperbaric oxygen therapy (HBOT) is one of the common clinical treatments, but adverse effects have hampered and limited the clinical application and promotion of hyperbaric oxygen therapy. A systematic review and meta-analysis of the adverse effects of hyperbaric oxygen therapy have conducted by our group to provide a theoretical basis for clinical treatment.

**Methods:**

Three electronic databases (PubMed, Web of Science, and The Cochrane Library) were comprehensively searched for randomized clinical trials (RCTs) from March 2012 to October 2022. Two reviewers independently screened titles and abstracts for eligibility and assessed the quality of the included studies. The meta-analysis was performed using RevMan 5.3.

**Results:**

A total of 24 RCTs involving 1,497 participants were identified. ① The HBOT group reported more adverse effects (30.11% vs. 10.43%, *p* < 0.05). ② The most frequent side effect of HBOT is ear discomfort (113 cases). ③ When the course of hyperbaric oxygen was >10 sessions, the incidence of adverse effects was higher than that of the control group; when the course of HBOT was ≤10 sessions, the adverse effects caused by hyperbaric oxygen were comparatively lower. ④ When the chamber pressure is above 2.0 ATA, the incidence of adverse effects is higher than that of the control group. While the chamber pressure is lower than 2.0 ATA, HBOT is relatively safe compared with the previous one.

**Conclusion:**

Hyperbaric oxygen therapy (HBOT) is more likely to cause adverse reactions when the chamber pressure is above 2.0 ATA. More attention should be paid to the possible occurrence of related adverse effects if the treatment course is >10 sessions.

**Systematic review registration:**

https://www.crd.york.ac.uk/PROSPERO/, identifier CRD42022316605.

## 1. Introduction

Hyperbaric oxygen therapy (HBOT), the treatment of a disease or medical condition by the inhalation of approximately 100% (at least 95%) medical grade oxygen at pressures between 1.2 and 3.0 atm absolute (ATA), has become a well-proven treatment modality for multiple conditions (1). Commonly, mild hyperbaric oxygen therapy is currently considered to be exposures delivered at pressures lower than 1.5 ATA. The clinical application of HBOT is gradually more popular and currently approved indications include air or gas embolism, acute thermal burn injury, carbon monoxide poisoning, central retinal artery occlusion, clostridial myositis and myonecrosis, decompression sickness, delayed radiation injury, idiopathic sudden sensorineural hearing loss, intracranial abscess, and necrotizing soft tissue infections. In addition to approved indications, further studies which demonstrate the potential applications and translation of HBOT in the field of inflammatory and systemic conditions, cancer, COVID-19, and other conditions are summarized (2).

During the application of HBOT, a few adverse effects have been identified. For instance, middle ear barotrauma, sinus and paranasal sinus barotrauma, ocular side effects, hypoglycemia, oxygen-induced seizures, and claustrophobia are basically well-identified adverse effects (3). However, systematic reviews and meta-analyses of the adverse effects of HBOT are still lacking since the occurrence of these adverse effects mentioned above could influence the application and promotion of HBOT. To fill the blank of this, a systematic review and meta-analysis of the adverse effects of HBOT have been conducted in this study to provide a theoretical basis for clinical treatment.

In other words, the research question for this systematic review can be summarized as follows:

Whether hyperbaric oxygen therapy causes more adverse effects or not, if compared with sham therapy or another intervention?

## 2. Methods

This systematic review was conducted according to the Preferred Reporting Items for Systematic Reviews and Meta-Analyses (PRISMA) guidelines (4). This review has been registered in PROSPERO (registered ID CRD42022316605).

### 2.1. Data sources and search strategies

Three electronic databases (PubMed, Web of Science, The Cochrane Library) were comprehensively searched for randomized controlled trials (RCTs) from March 2012 to October 2022 by two authors independently, without any language restrictions. Taking PubMed as an example, the following search terms were used for study retrieval: ((((((((((Hyperbaric Oxygenations) OR (Oxygenations, Hyperbaric)) OR (Hyperbaric Oxygen Therapy)) OR (Hyperbaric Oxygen Therapies)) OR (Oxygen Therapies, Hyperbaric)) OR (Oxygen Therapy, Hyperbaric)) OR (Therapies, Hyperbaric Oxygen)) OR (Therapy, Hyperbaric Oxygen)) OR (Oxygenation, Hyperbaric)) OR (HBO)) OR (HBOT).

### 2.2. Study selection

Two investigators reviewed and selected the studies according to the predetermined criteria. All potentially relevant articles were retrieved from the databases for the assessment of their full text based on titles and abstracts. Only RCTs were included in the analysis. Case–control studies, case series, and case reports were not considered. All participants in the treatment group received HBOT alone or in combination with other therapeutic approaches, with no restriction on age, gender, race, and severity of disease. While some criteria show that a certain group supposes to be excluded from the study, studies on mild hyperbaric oxygen therapy were excluded; patients in the control group received placebo or other treatments except for HBOT; studies with retrospective nature, irrelevant topics, no controls, duplicated data, or insufficient data were also excluded. The results include the adverse effects of HBOT.

### 2.3. Data extraction and quality assessment

A pre-defined Excel spreadsheet was utilized for data collection. Extracted information includes the first author’s name, year of publication, age, sample size, interventions, follow-up, and adverse events. The first or correspondence author is directly contracted by e-mail for insufficient or ambiguous data. Discrepancies were resolved by group discussion.

Two authors evaluated the risk of bias with regard to adverse event outcomes by using the tool recommended by the Cochrane Collaboration Handbook. Each study was categorized into “low,” “unclear,” and “high” risk of bias by two reviewers, based on the following domains: random sequence generation, allocation concealment, blinding to participants, researchers and outcome evaluators, incomplete data, selective outcome reporting, and other sources of bias.

### 2.4. Statistical analysis

Statistical analysis was performed by Review Manager 5.3. For each included study, we calculated the risk ratio and 95% confidence interval (95% CI) for the incidence rate in the intervention arm compared with that of the control, based on the reported number of events and sample size. We used the I^2^ index to examine heterogeneity across trials for each outcome. A fixed effect model was utilized for meta-analysis if *I*^2^ < 25% or *p* > 0.10. Otherwise, a random effect model was used (*I*^2^ > 25% or *p* < 0.10). The significance was accepted at *p* < 0.05. We conducted subgroup analysis by different control groups, different adverse events, different treatment courses, different chamber pressures, and different types of diseases. For subgroup analysis of different adverse effects, if a particular adverse effect was reported in no more than two studies, the adverse effect would be included in the “other adverse effects”; if the study mentioned the adverse event as a barotrauma but did not mention that the barotrauma site, it was not included in the subgroup analysis. For subgroup analyses of different types of diseases, if one disease was evaluated in no more than two studies, it would not be included in the subgroup analysis.

## 3. Results

### 3.1. Summary of the included studies

**Figure 1:**
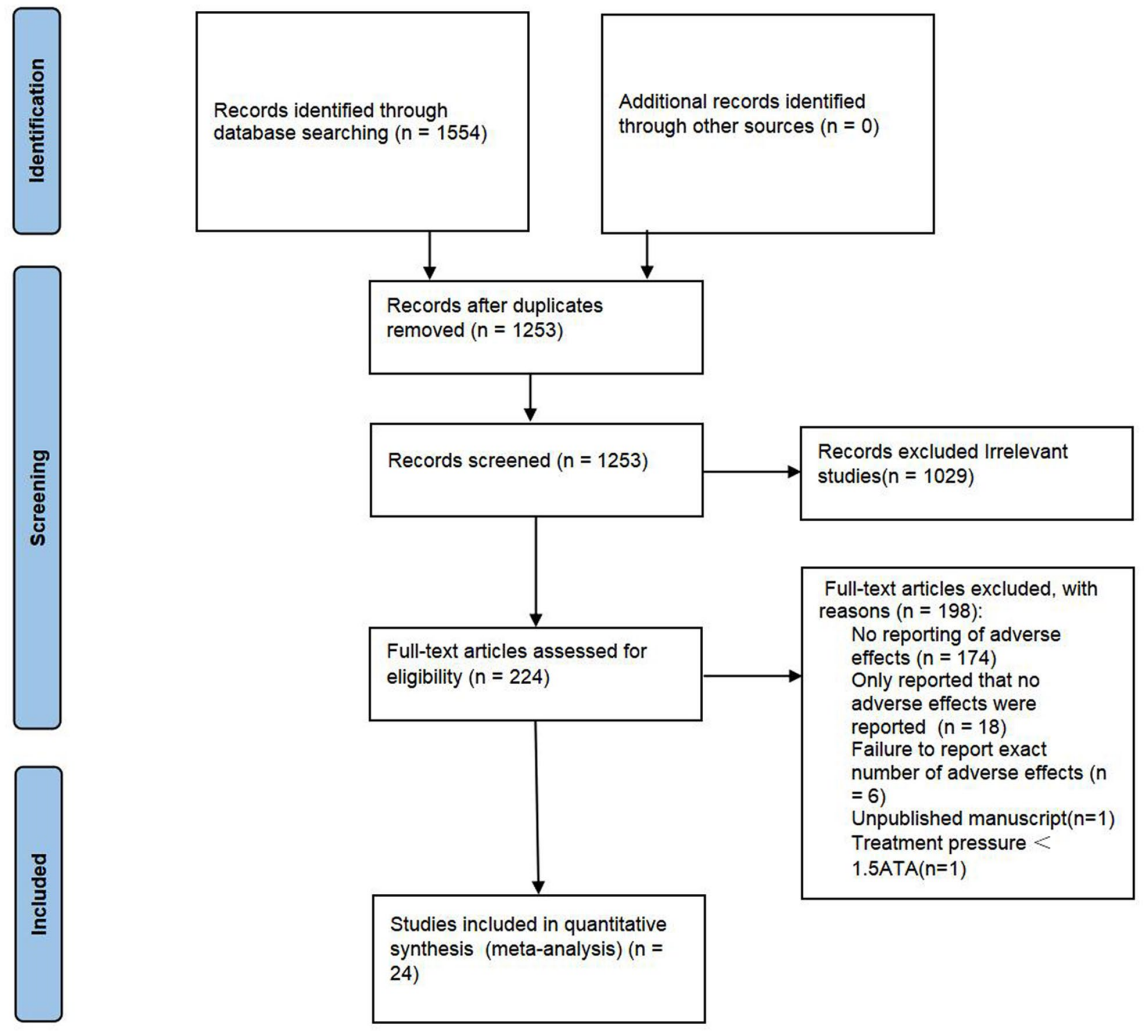
PRISMA flow diagram.

A total of 1,554 articles were identified. In total, 301 duplications and another 1,029 records which are considered as ineligible after scrutinizing the title and abstract are removed. Thus, 129 full-text articles were further assessed for eligibility. As shown in Figure 1, studies with no reporting of adverse effects (*n* = 174), only report that no adverse events were reported (*n* = 18), failure to report the exact number of adverse events (*n* = 6), unpublished manuscript (*n* = 1), and treatment pressure <1.5ATA (*n* = 1) are excluded. Finally, 24 RCTs (5–27) involving 1,497 participants (797 in the HBOT group and 700 in the control group) were included for meta-analysis.

**Figure 2:**
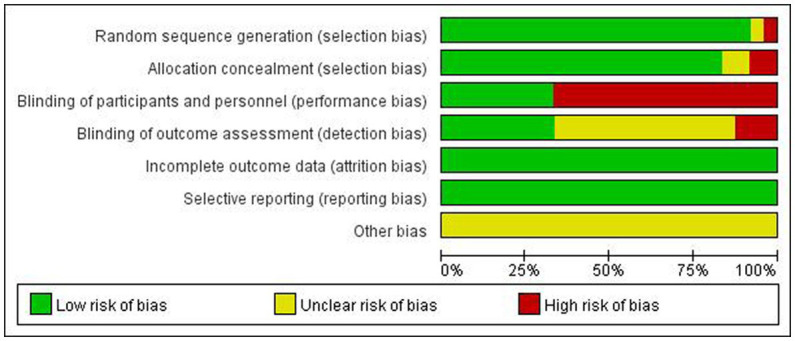
Methodological quality summary: review authors’ judgments about each methodological quality item for each included study.

Detailed characteristics of included trials are presented in Table 1. All studies were published from 2012 to 2022. The ages of participants range from 5 to 70 years. Hyperbaric oxygen therapy was explicitly described by authors in 13 of the trials, including chamber pressures and treatment courses, while seven of them specified the rate of compression. Diseases involved in the studies included cerebral palsy, childhood autism, stroke, sudden sensorineural hearing loss, fibromyalgia syndrome, persistent postconcussion symptoms, diabetes with non-healing ulcers of the lower limb, chronic bowel dysfunction after pelvic radiotherapy, prostate cancer, adhesive postoperative small bowel obstruction, chronic venous leg ulcers, radiation-induced cystitis, osteoradionecrosis, mild traumatic brain injury, central airway stenosis after lung transplantation, post-traumatic stress disorder, and chronic non-healing ulcer. In all trials, the treatment course ranged from 7 to 60 sessions with the chamber pressures and control group being 1.5–2.5ATA and 1.03–2.2ATA, respectively. The adverse effects mentioned in the studies included ear discomfort, sinus pain, ocular side effects, seizure, claustrophobia, chest pain, gastrointestinal reaction, headache, fatigue, and congestive heart failure. Figure 2 shows the evaluated risk of bias.

**Table 1:** Details of HBOT studies included in the performance meta-analysis.

Study ID	Sample size		Age (years)		Disease	Intervention		Course (session)	Adverse events	
	T	C	T	C		T	C		T	C
Lacey2012 (28)	24	22	6.3 ± 1.3	5.2 ± 2.0	Cerebral palsy	100% oxygen at a pressure (or depth) of 1.5ATA	Room air (21% oxygen) at 1.5ATA	40	Ear pain (7)	Ear pain (8)
Sampanthavivat2012 (5)	29	29	6.10	5.67	Childhood autism	100% oxygen at a pressure (or depth) of 1.5ATA	Room Air (21% oxygen) at 1.15ATA	20	Minor-grade ear barotrauma (11)	Minor-grade ear barotrauma (3)
Chen2013 (6)	33	32	60.3 ± 9.3	60.5 ± 9.5	Progressive cerebral infarction	100% oxygen at a pressure (or depth) of 1.5ATA	Conventional treatment	14	Ear pain (1); gastrointestinal reaction (1)	Rash (1)
Efrati2013 (7)	59	29	61 ± 12	63 ± 6.3	Stroke	90 min each, 100% oxygen at 2ATA	Conventional treatment	40	Mild–moderate barotrauma of the middle ear (6)	No
Cvorovic2013 (8)	25	25	53.6 ± 15.5	47.3 ± 10.8	Sudden sensorineural hearing loss	100% oxygen at a pressure (or depth) of 2.0ATA	Conventional treatment	20	Serous otitis media (3)	No
Efrati2015 (9)	48	26	50.4 ± 10.9	48.1 ± 11.1	Fibromyalgia syndrome	90 min, 100% oxygen at 2ATA	Conventional treatment	40	Mild barotrauma (13)	No
Miller2015 (10)	24	23	32.5	31.4	Persistent postconcussion symptoms	100% oxygen at a pressure (or depth) of 1.5ATA	Room Air (21% oxygen) at 1.2ATA	40	Middle ear pain (1); Inner ear barotrauma (3); Tooth pain (1); Onset migraine headache (2); Transient worsening of myopia (1)	Middle ear pain (1); change in headaches frequency (1); Claustrophobia/anxiety (1); Sinus pain (3)
Fedorko 2016 (11)	49	54	61	62	Diabetes with nonhealing ulcers of the lower limb	100% oxygen at a pressure (or depth) of 2.4ATA	Room air (21% oxygen)at 1.2ATA	30	Barotraumas (3); Unable to equalize ears (4); Visual changes (4); Anxiety, chest pain (2); Nausea (3); Hypoglycemia (4); Wound infection (2); Pain postmyringotomy (1); Congestive heart failure (1)	Barotraumas (3); Visual changes (3); Nausea (1); Hypoglycemia (1);
Glover2016 (12)	53	28	62.3	62.0	Chronic bowel dysfunction after pelvic radiotherapy	90 min, 100% oxygen at 2ATA	Room Air (21% oxygen) at 1.3ATA	40	Myopia (16); Fatigue (2); Ear pain or barotrauma (15)	Myopia (3); Fatigue (3); Ear pain or barotrauma (6)
Chiles2018 (13)	40	43	40–65	40–65	Prostate cancer	100% oxygen at a pressure (or depth) of 2.2ATA	Room Air (21% oxygen) at 2.2ATA	10	Immediate urine leak (1); Ear pressure (2); Hypertension (1); Myopia (1); Urinary tract infection (1); Incontinence (1)	Ear pressure (1); Meatal stenosis (1)
Fukami2018 (14)	33	40	66	62	Adhesive postoperative small bowel obstruction	100% oxygen at a pressure (or depth) of 2.0ATA	Conservative treatment	7	Mild earache (1)	No
Santema2017 (15)	53	56	67.6	70.6	Ischemic lower extremity ulcers in patients with diabetes	100% oxygen at a pressure (or depth) of 2.4ATA	Standard care	40	Oxygen induced seizure (1); Barotraumatic perforation of the tympanic membrane (1); Inability to equalize the pressure of the middle ear (3)	No
Thistlethwaite2018 (16)	15	15	70	70	Chronic venous leg ulcers	100% oxygen at a pressure (or depth) of 2.4ATA	Room Air (21% oxygen) at 1.2ATA	30	Otic barotraumas (2)	No
Oscarsson2019 (17)	41	38	64.0	64.8	Radiation-induced cystitis	100% oxygen at a pressure (or depth) of 2.5ATA	Standard care	30–40	Ear pain (6); myopia (5); Barotrauma (4)	Cardiac failure (1)
Shaw2019 (18)	47	53	58.3	58.2	Osteoradionecrosis	100% oxygen at a pressure (or depth) of 2.4ATA	Conventional treatment	30	Hearing impaired (1); Ear barotrauma (4); Eye disorders (1); Fatigue (1); Chest wall pain (1); seizure (1); Epistaxis (1); Hypotension (1)	No
Weaver2019 (19)	60	58	34.8(BIMA)/32.5(HOPPS)	30.8(BIMA)/31.4(HOPPS)	Mild traumatic brain injury	100% oxygen at a pressure (or depth) of 1.5ATA	Room Air (21% oxygen) at 1.2ATA	40	Ear discomfort (15); Sinus pain (5); Dizziness (1); Vertigo (1); Headache (1); Somnolent (1); Dyspnea (2); Hyperventilation (1); Eye disorders (1); Anxiety (1)	Ear discomfort (6); Sinus pain (4); Dizziness (1); Headache (2); Somnolent (1); Eye disorders (2); Vertigo (1)
Hadanny2020 (20)	30	33	70.68 ± 3.64	68.81 ± 3.34	Healthy older adults	100% oxygen at a pressure (or depth) of 2.0ATA	Conventional treatment	60	Mild middle ear barotrauma (4); Visual acuity changes (15)	Visual acuity changes (10)
Harch2020 (21)	50	27	42.7 ± 10.7	42.3 ± 11.2	Mild traumatic brain injury	100% oxygen at a pressure (or depth) of 1.5ATA	Conventional treatment	40	Fatigue (2); Mild reversible middle Ear barotrauma (1); A multiply previously perforated tympanic membrane (1)	No
Schiavo2020 (22)	13	11	62 ± 11	61 ± 10	Stroke	100% oxygen at a pressure (or depth) of 2.0ATA	Conventional treatment	40	Middle-ear barotrauma (4); Chest pain (1)	No
Curtis2021 (23)	17	8	45.7 ± 14.2	51.8 ± 14.5	Fibromyalgia	100% oxygen at a pressure (or depth) of 2.0ATA	Conventional treatment	40	Mild middle-ear barotrauma (3); New-onset myopia (4)	No
Kraft2021 (24)	10	10	59.7	54.5	Central airway stenosis after lung transplantation	100% oxygen at a pressure (or depth) of 2.0ATA	Standard care	20	Claustrophobia (1)	No
Doenyas-Barak2022 (25)	14	15	39.3 ± 8.1	32.4 ± 9.2	Post-traumatic stress disorder	100% oxygen at a pressure (or depth) of 2.0ATA	Conventional treatment	60	Middle ear barotrauma (7)	No
Hadanny2022 (26)	15	10	11.99 ± 2.32	11.00 ± 2.32	Post-concussion syndrome	100% oxygen at a pressure (or depth) of 1.5ATA	Room Air (21% oxygen) at 1.03ATA	60	Ear pain (2); Mild–moderate barotrauma (9); Headache (2)	Ear pain (5); Mild–moderate barotrauma (4)
Kaur2012 (27)	15	15	46.9 ± 11.8	47.4 ± 12.5	Chronic nonhealing ulcer	100% oxygen at a pressure (or depth) of 2.5ATA	Conventional treatment	30	Ear discomfort/pain (3); Claustrophobia (2); Headache (1); Tinnitus (1)	No

### 3.2. Meta-analysis results

#### 3.2.1. Incidence of adverse effects

**Figure 3:**
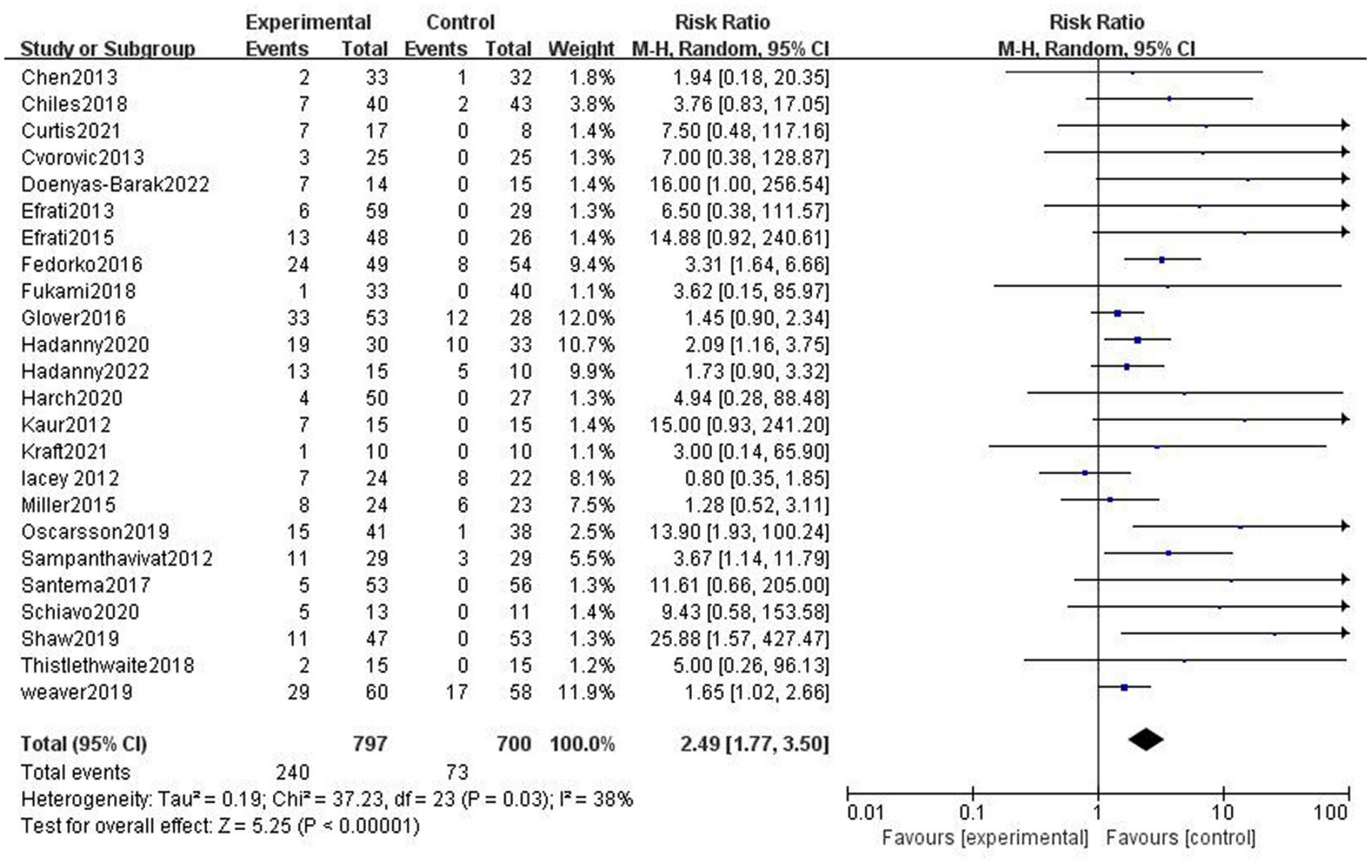
Analysis 1.1: HBOT versus any control group, any adverse event. CI, confidence interval; df, degrees of freedom; M–H, Mantel–Haenszel method of meta-analysis; P, probability; Z, *Z*-score (standard score).

There was heterogeneity between the studies (*p* = 0.03, *I*^2^ = 38%); therefore, a random effect model was performed. It turns out that the incidence of AEs in the HBOT group was higher than that in the control group (30.11% vs. 10.43%, RR = 2.89, 95%CI:1.77–3.50, *p* < 0.05; Figure 3).

#### 3.2.2. Subgroup analysis

##### 3.2.2.1. Effect of different control groups

**Figure 4:**
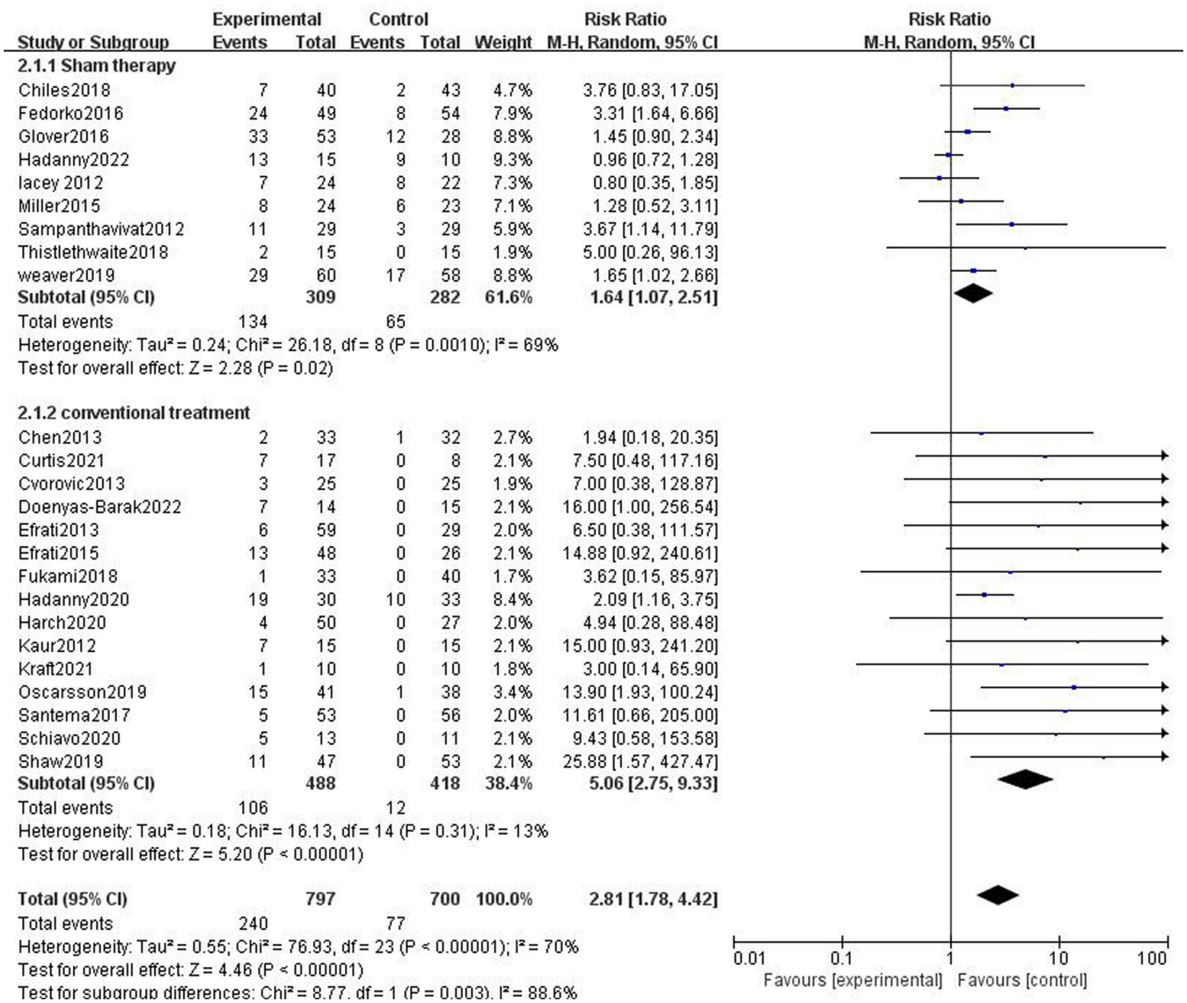
Analysis 2.1: HBOT versus sham therapy and conventional treatment, any adverse event. CI, confidence interval; df, degrees of freedom; M–H, Mantel–Haenszel method of meta-analysis; P, probability; Z, *Z*-score (standard score).

In nine studies, participants in the control group received sham therapy. Compared with patients in the control group, patients in the HBOT group were more likely to have AEs (43.37% vs. 23.05%, RR = 1.88, 95%CI:1.07–2.51, *p* = 0.02; Figure 4), with high heterogeneity (*p* = 0.0010, *I*^2^ = 69%). In 15 studies, patients in the control group received conventional treatment, and it turns out that the incidence of AEs was higher in the HBOT group than in the control group (21.93% vs. 2.87%, RR = 7.57, 95%CI:2.75–9.33, *P* < 0.00001; Figure 4), with low heterogeneity (*p* = 0.31, *I*^2^ = 13%).

##### 3.2.2.2. Effect of different adverse events

Table 2 summarizes the results of the subgroup analysis of different adverse events. We found significantly increased risk ratios with HBOT compared with the control group for two specific adverse events, such as ear discomfort and ocular side effects.

**Table 2:** Results of subgroup analysis of different adverse events.

Adverse events	No. of trails	*P*	RR	95%CI	Test of Heterogeneity	
					P	*I*^2^ %
Ear discomfort	22	<0.01	3.38	1.61–4.41	0.09	30
Sinus pain	3	0.77	0.88	0.32–2.29	0.28	21
Ocular side effects	9	<0.01	2.37	1.29–3.32	0.83	0
Seizure	2	0.30	—*	0.35–30.92	0.98	0
Claustrophobia	3	0.45	2.94	0.40–7.94	0.42	0
Chest pain	3	0.14	—*	0.64–22.13	0.94	0
Gastrointestinal reaction	2	0.21	4.22	0.15–19.60	0.95	0
Headache	4	0.47	1.86	0.46–5.28	0.70	0
Fatigue	3	0.92	1.20	0.29–3.10	0.31	15
Congestive heart failure	2	0.99	1.02	0.15–6.77	0.30	6

“*”: The incidence of this adverse effect in the control group was 0. The relative risk could not be calculated.

1. Ear discomfort: A total of 22 studies (5, 7, 8, 10–14, 17, 20, 21, 23, 25, 28) reported ear discomfort. The risk of ear discomfort was increased in participants treated with HBOT compared with either sham therapy or other conventional treatments (RR = 3.38, 95%CI:1.61–4.41, *P* < 0.01), with heterogeneity (*p* = 0.09, *I*^2^ = 30%).
2. Sinus pain: Three studies (10, 18, 19) reported sinus pain. The incidence of sinus pain was higher in the HBOT group than in the control group, with low heterogeneity (*p* = 0.28, *I*^2^ = 21%). The difference was not statistically significant (RR = 0.88, 95%CI:0.32–2.29, *p* > 0.05).
3. Ocular side effects: Nine studies (10–13, 17–20, 23) reported ocular side effects. The risk of ocular side effects was increased in participants treated with HBOT compared with either sham therapy or other conventional treatments (RR = 2.37, 95%CI:1.29–3.32, *P*<0.05), with no heterogeneity (*p* = 0.83, *I*^2^ = 0%).
4. Seizure: Two studies (15, 18) reported seizure. The incidence of seizure was higher in the HBOT group than in the control group, with no heterogeneity (*p* = 0.98, *I*^2^ = 0%). The difference was not statistically significant (95%CI:0.35–30.92, *p* > 0.05).
5. Claustrophobia: Three studies (10, 24, 27) reported claustrophobia. The incidence of claustrophobia was higher in the HBOT group than in the control group, with no heterogeneity (*p* = 0.42, *I*^2^ = 0%). The difference was not statistically significant (RR = 2.94, 95%CI:0.40–7.94, *p* > 0.05).
6. Chest pain: Three studies (11, 18, 22) reported chest pain. The incidence of chest pain was higher in the HBOT group than in the control group, with no heterogeneity (*p* = 0.94, *I*^2^ = 0%). The difference was not statistically significant (95%CI:0.64–22.13, *p* > 0.05).
7. Gastrointestinal reaction: Two studies (6, 11) reported gastrointestinal reaction. The incidence of gastrointestinal reaction was higher in the HBOT group than in the control group, with no heterogeneity (*p* = 0.95, *I*^2^ = 0%). The difference was not statistically significant (RR = 4.22, 95%CI:0.15–19.60, *p* > 0.05).
8. Headache: Four studies (10, 19, 26, 27) reported headache. The incidence of headache was lower in the HBOT group than in the control group, with no heterogeneity (*p* = 0.70, *I*^2^ = 0%). The difference was not statistically significant (RR = 1.86, 95%CI: 0.46–5.28, *p* > 0.05).
9. Fatigue: Three studies (12, 18, 21) reported fatigue. The incidence of fatigue was higher in the HBOT group than in the control group, with no heterogeneity (*p* = 0.31, *I*^2^ = 15%). The difference was not statistically significant (RR = 1.20, 95%CI:0.29–3.10, *p* > 0.05).
10. Congestive heart failure: Two studies (11, 17) reported congestive heart failure. The incidence of congestive heart failure was higher in the HBOT group than in the control group, with no heterogeneity (*p* = 0.30, *I*^2^ = 6%). The difference was not statistically significant (RR = 1.02, 95%CI:0.15–6.77, *p* > 0.05).
11. Other AEs: Other AEs caused by the HBOT included hypoglycemia, vertigo, tooth pain, somnolence, anxiety, dyspnea, hyperventilation, urinary incontinence, urinary tract infection, hypotension, and hypertension, as shown in Table 3.

**Table 3:** Other adverse events during HBOT.

Adverse events	Study ID	HBOT		Control	
		Events	Total	Events	Total
Hypoglycemia	Fedorko2016 (11)	4	49	1	54
Dizziness/ vertigo	Weaver2019 (19)	2	60	2	58
Tooth pain	Miller2015 (10)	1	24	0	23
Somnolent	Weaver2019 (19)	1	60	1	58
Anxiety	Weaver2019 (19)	1	60	0	58
Dyspnea	Weaver2019 (19)	2	60	0	58
Hyperventilation	Weaver2019 (19)	1	60	0	58
Incontinence	Chiles2018 (13)	2	40	0	43
Urinary tract infection	Chiles2018 (13)	1	40	0	43
Meatal stenosis	Chiles2018 (13)	0	40	1	43
Hypotension	Shaw2019 (18)	1	47	0	53
Hypertension	Chiles2018 (13)	1	40	0	43

##### 3.2.2.3. Effect of different treatment courses

**Figure 5:**
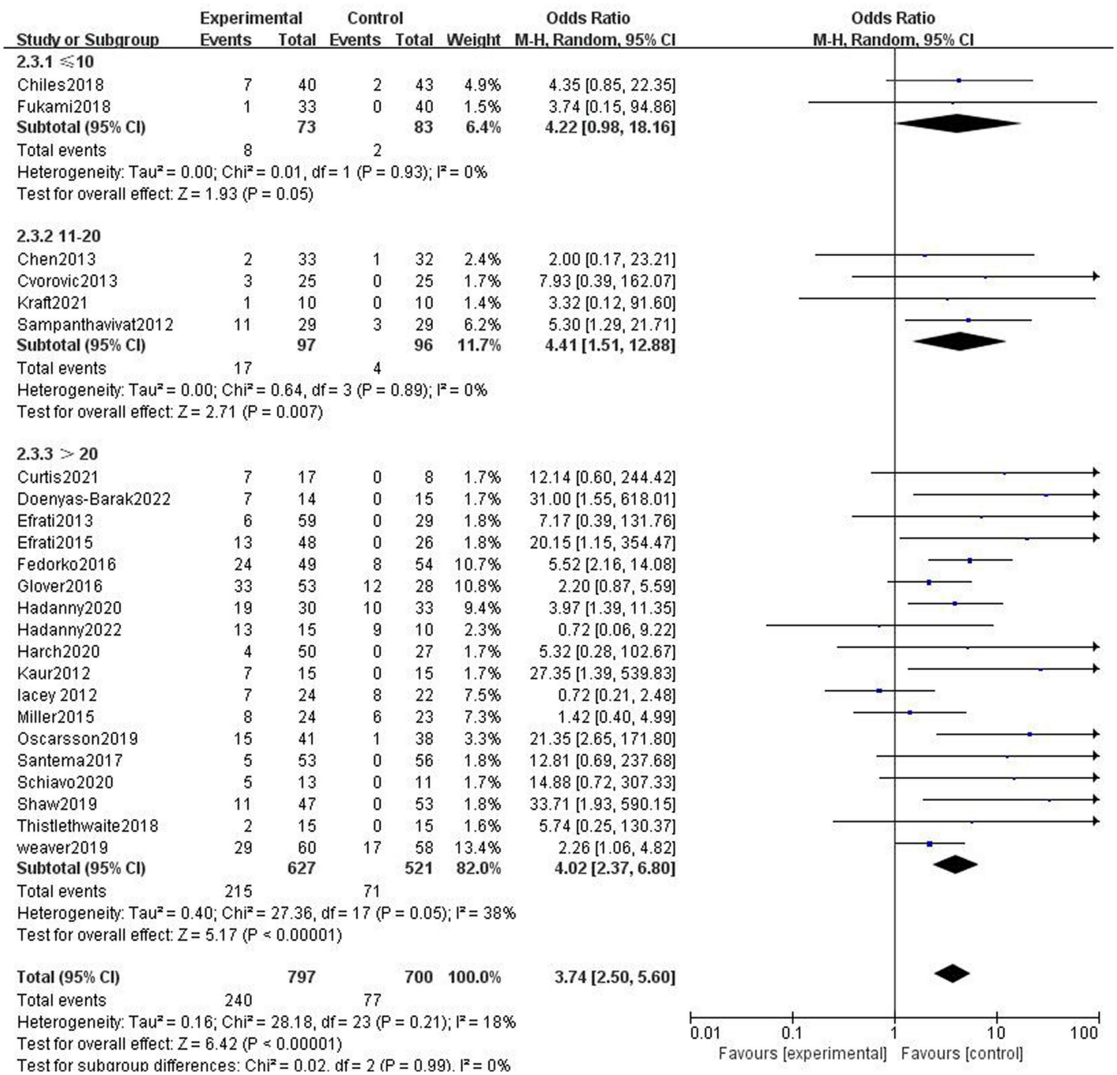
Analysis 2.3: ≤10 sessions, 11–20 sessions, >20 sessions of HBOT versus any control group, any adverse event. CI, confidence interval; df, degrees of freedom; M–H, Mantel–Haenszel method of meta-analysis; P, probability; Z, *Z*-score (standard score).

In two studies, participants in the HBOT group received ≤10 sessions of HBOT. The incidence of AEs was higher in the HBOT group than in the control group, with no heterogeneity (*p* = 0.93, *I*^2^ = 0%). The difference was not statistically significant (RR = 4.54, 95%CI: 0.98–18.16, *p* = 0.05). In four studies, participants in the HBOT group received 11–20 sessions of HBOT. Compared with patients in the control group, patients in the HBOT group were more likely to have AEs (RR = 4.20, 95%CI:1.51–12.88, *p* = 0.007), with no heterogeneity (*p* = 0.89, *I*^2^ = 0%). In 19 studies, patients in the HBOT group received >20 sessions of HBOT. Compared with patients in the control group, patients in the HBOT group were more likely to have AEs (RR = 2.52, 95%CI:2.37–6.80, *P* < 0.05; Figure 5), with a heterogeneity (*p* = 0.05, *I*^2^ = 38%).

##### 3.2.2.4. Effect of different chamber pressures

**Figure 6:**
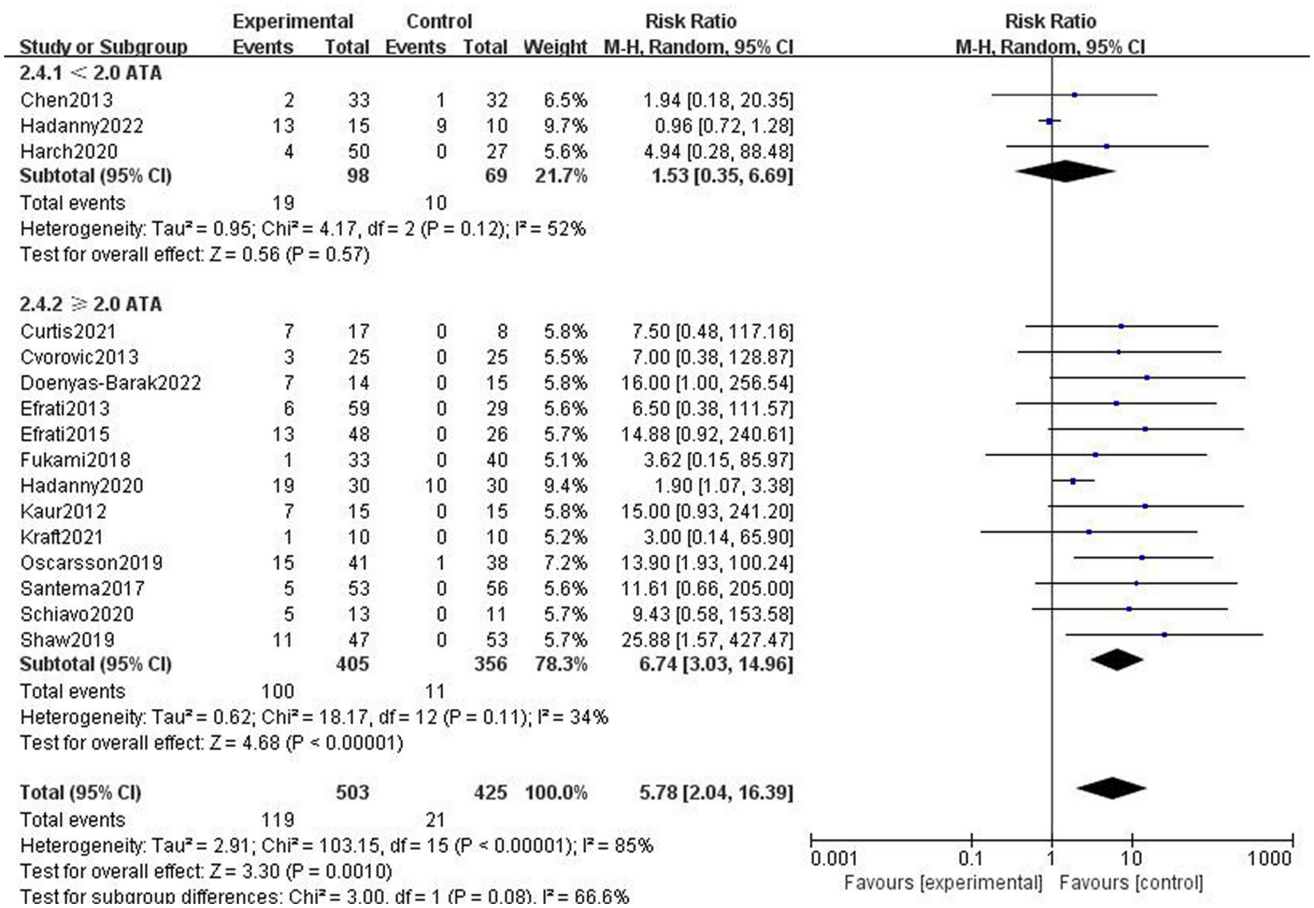
Analysis 2.4: <2.0 ATA, ≥2.0 ATA chamber pressures of HBOT versus any control group, any adverse event. CI, confidence interval; df, degrees of freedom; M–H, Mantel–Haenszel method of meta-analysis; P, probability; *Z, Z*-score (standard score).

The studies were divided into two subgroups according to chamber pressure. Since the results demonstrated heterogeneity in the two subgroups, a random effect model was applied to analyze the results. Due to the high chamber pressure in some of the control groups, the studies with sham therapy control groups were not included in this subgroup analysis. The incidence of adverse effects was higher in the HBOT group than in the control group for subgroups with a chamber pressure of ≥2.0 ATA, which represents statistically significant differences in the results (RR = 7.99, 95%CI:3.03–14.96, *P* < 0.00001; Figure 6). The difference in the incidence of adverse effects between the hyperbaric and control groups in the subgroup with the pressure of <2.0 ATA was not statistically significant (*R* = 1.34, 95% CI: 0.35–6.69, *p* > 0.05; Figure 6).

##### 3.2.2.5. Effect of different types of diseases

**Figure 7:**
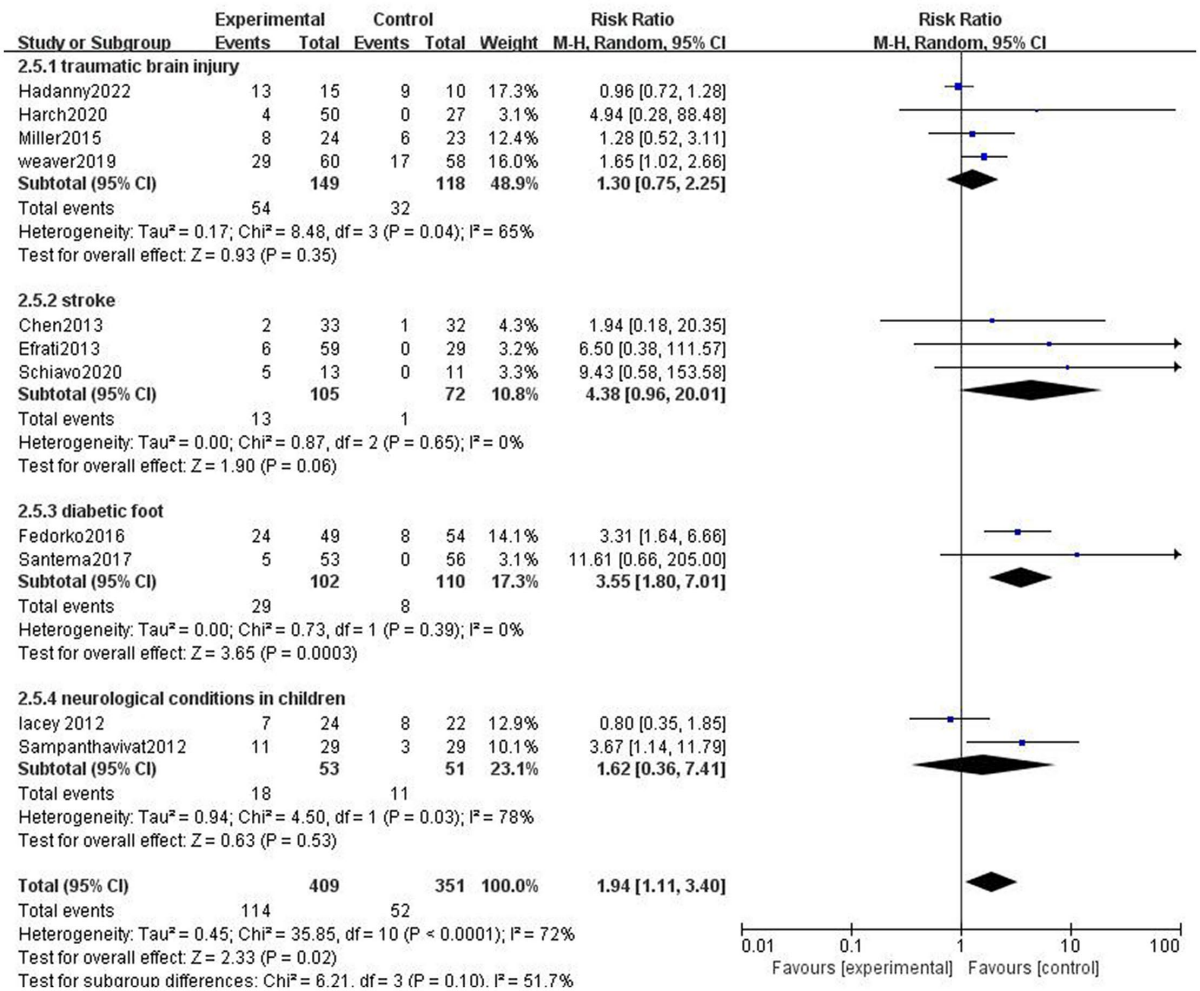
Analysis 2.5: HBOT in traumatic brain injury, stroke, diabetic foot, and neurological conditions in children, any control group, any adverse event. CI, confidence interval; df, degrees of freedom; M–H, Mantel–Haenszel method of meta-analysis; P, probability; *Z*, *Z*-score (standard score).

The studies were divided into traumatic brain injury subgroup, stroke subgroup, diabetic foot subgroup, and neurological conditions in children (cerebral palsy and autism). Adverse effects were more frequent in the HBOT group than in the control group in the diabetic foot subgroup (Figure 7).

## 4. Discussion

The results of this meta-analysis demonstrated that the incidence of adverse effects was higher in the hyperbaric group, regardless of whether the control group was a sham or conventional treatment group. The adverse effects of HBOT with a statistically significant difference from the control/sham group are ear discomfort (RR = 3.38, 95%CI:1.61–4.41, *P* < 0.01, with heterogeneity; *p* = 0.09, *I*^2^ = 30%) and ocular side effects (RR = 2.37, 95%CI: 1.29–3.32, *P* < 0.05, with no heterogeneity; *p* = 0.83, *I*^2^ = 0%). Most of the adverse effects of hyperbaric oxygen are mild and self-limiting, the most common of which is middle ear barotrauma, an adverse effect that can be prevented by ongoing teaching of middle ear clearing techniques and appropriate compression rates (3).

The adverse effects of HBOT can be divided into two categories: adverse effects of pressure and adverse effects of oxygen. The adverse effect of pressure includes barotrauma, which can affect any closed, air-filled cavity (including but not limited to the ears, sinus, teeth, lungs, and bowel). The adverse effects of oxygen can further be subdivided into three categories as follows: pulmonary, neurologic, and ophthalmologic (29). Patients in the sham therapy group were mostly treated with normobaric or hyperbaric room air. In Chiles2018 (13) and Lacey2012 (28), chamber pressures in the control groups were consistent with that of the HBOT groups. The incidence of ear discomfort in these studies was found to be similar in the HBOT groups (14.06%) and the control groups (13.85%). Therefore, the factor of injury for ear discomfort may originate more from pressure rather than oxygen toxicity.

Both ear and ocular adverse effects were more frequent in the HBOT group than in the control group, while the differences in the incidence of the remaining several adverse effects were not statistically significant. It might be caused by several reasons as follows: the exclusion of this adverse effect as a contraindication; the small number of cases involving this adverse effect; and the relatively mild clinical manifestation of the adverse effect, which failed to attract the attention of the participants.

Data analysis indicated that a lower incidence of claustrophobia was found in the HBOT group than in the control group. There is a possibility that this is due to the fact that the control group in Miller2015 (10) was a sham therapy group in which participants would also enter the chamber; in parallel, claustrophobia is one of the contraindications to HBOT, while few people have previous claustrophobia which is not detected. Claustrophobia may be managed with coaching and anxiolytic medications. Intolerance of a monoplace chamber may warrant referral to the closest multiplace chamber facility (3).

Some adverse effects may also be related to the patient’s health condition rather than the HBOT, for instance, participants in Chiles2018 (13) experienced adverse effects in the form of urinary incontinence and urinary tract infections, which may be related to undergoing radical prostate cancer surgery. Similarly, cardiovascular adverse effects show a similar pattern. The onset of congestive heart failure in the patients of Fedorko2016 (11) and Oscarsson2019 (17) in this study may also be associated with the participants’ health conditions. With regard to the mechanisms of congestive heart failure, a study by Weaver et al. (30) suggested that hyperbaric oxygen therapy could increase left ventricular (LV) afterload, LV filling pressures, and oxidative myocardial stress and decrease LV compliance by oxygen radical-mediated reduction in nitric oxide, alter cardiac output between the right heart and left heart, and induce bradycardia with concomitant LV dysfunction. Therefore, caution should be exercised in the use of hyperbaric oxygen therapy in patients with heart failure or reduced cardiac ejection fractions, and we recommend to ensure that the patient’s cardiac function is in pharmacological compensatory before initiating HBO therapy. With regard to the effect of HBOT on blood pressure, most studies report an increase in blood pressure. Al-Waili et al. (31) pointed out that hyperbaric oxygen can cause hypertension, which was seen in one case of hypertension in the hyperbaric group in Chiles2018 (13). A different result, however, was seen in Shaw 2019 (18), where there was one case of hypotension, but the study did not mention its cause.

Our results revealed that at a course of >10 sessions, the incidence of adverse effects was greater than that of the control group. When the treatment course was ≤10 sessions, the adverse effects were relatively low. The main adverse effects that warranted attention were ear adverse effects, such as ear pain (13, 14). The outcome implies that the course of HBOT is a major influencing factor for the adverse effects, but it does not necessarily mean that the treatment course should be shortened to less than 10 sessions. It is suggested that more attention should be paid to the possible occurrence of related adverse effects and discomforts from observations or asking patients directly instead of shortening the treatment course to less than 10 sessions. Afterward, appropriate protective measures should be taken based on the observation.

In the present study, the results indicated that patients who received HBOT at chamber pressures above 2.0 ATA had a higher incidence of adverse effects than the control one. The incidence of adverse effects is relatively low, with a chamber pressure below 2.0 ATA. The adverse effects to be cautioned about are mainly ear discomfort, ocular side effects, headache, sinus barotrauma, etc. (6, 10, 19, 21, 28). Ajayi et al. (32) suggested that the incidence of adverse effects of HBOT at a chamber pressure of 2.0 ATA was similar to that of 2.4 ATA. As for the incidence of seizures, Marvin et al. (33) noted that there was a statistically significant difference in seizure between the different pressures. They demonstrated a statistically significant increased risk of seizure with increasing treatment pressure. Research conducted by Resanovic et al. and MijajlovicI et al. (34, 35), however, suggested that HBOT with chamber pressures below 3.0 ATA could rarely cause adverse effects. It is probably related to the fact that, in general, the adverse effects of HBOT are mild and mostly self-limiting (3), as such many patients do not report even though the adverse effects occur.

It has also been suggested that the incidence of adverse effects relates to different time intervals and rates (slope) of compression (36). Nevertheless, subgroup analyses were not performed since fewer of the studies explicitly described time interval and rate of compression and did not include them as categorical or control factors, which may affect the accuracy of the data analysis. Seven of the included studies (6, 8, 13, 14, 16, 20, 28) specify the rate of compression, but valid data statistics could not be performed as the rate of compression in the control group was not mentioned. In addition, nine studies (11–13, 16, 20–23, 25) reported time intervals. Owing to the 5-min time interval in most of the studies and the 0-min interval in only one study, it was not feasible to group the studies for subgroup analysis.

The results of this study revealed that the incidence of adverse effects was higher in patients with diabetic foot when receiving HBOT. Particular attention is necessary for the hypoglycemic occurrence in diabetics receiving HBOT. It has been documented that in diabetics, undergoing HBOT, severe hypoglycemia is rare and occurs more frequently in type 1 diabetes. Pre-HBOT glucose values may be used to predict subsequent hypoglycemia and reduce the need for routine glucose monitoring during and after HBOT (37). Fedorko2016 (11), a study of diabetics with non-healing ulcers of the lower limb, identified an occurrence of hypoglycemia in four of the 61 patients in the HBOT group.

Within children with neurological disorders, adverse effects regarding hyperbaric oxygen therapy did not differ significantly from controls, probably due to the similar pressure in both the HBOT and control groups in Lacey2012 (28).

Limitations also exist in this study. The small number of cases of partial adverse effects during subgroup analysis may have an implication on the results of the data analysis, especially when the heterogeneity between these small numbers of studies is relatively high. Exclusion as a contraindication resulted in a significant reduction in the incidence of some adverse reactions, such as claustrophobia, leading to no statistical significance of the difference in the incidence of this adverse effect between the HBOT and control groups. Comorbidities (fever, cold, cardiovascular disease, epilepsy in therapy, and others), hyperbaric chamber type (single-seat, multi-seat), and breathing system (mask, hood) have a significant influence on the frequency of adverse events. These important variables are not reported in this study.

In summary, the main adverse effects of HBOT are ear discomfort (e.g., middle ear barotrauma, ear pain, etc.) and ocular side effects (e.g., myopia, hyperopia, etc.). HBOT is more likely to cause adverse reactions when the chamber pressure is above 2.0 ATA. More attention should be paid to the possible occurrence of related adverse effects when the patients will receive more than 10 sessions of HBOT. However, it should be noted that the above views are mainly based on literature reviews. In clinical practice, the experience and seriousness of the therapist (including medical assistant) may affect the occurrence of side effects.

## Data availability statement

The original contributions presented in the study are included in the article/supplementary material, further inquiries can be directed to the corresponding author.

## Author contributions

YYZ, YJZ, and YJ contributed to conception and design of the study. YJZ organized the database. YYZ performed the statistical analysis and wrote the first draft of the manuscript. YYZ, YJZ, YJ, DM, and TW wrote sections of the manuscript. All authors contributed to manuscript revision, read, and approved the submitted version.

## Funding

This work was supported by the Key Research & Development Program of Jiangsu Province (grant No. BE2021012-4).

## Conflict of interest

The authors declare that the research was conducted in the absence of any commercial or financial relationships that could be construed as a potential conflict of interest.

## Publisher’s note

All claims expressed in this article are solely those of the authors and do not necessarily represent those of their affiliated organizations, or those of the publisher, the editors and the reviewers. Any product that may be evaluated in this article, or claim that may be made by its manufacturer, is not guaranteed or endorsed by the publisher.
